# Semi-automated rubrics for evidence-based medicine assessment: a case report on grading time reduction

**DOI:** 10.5195/jmla.2026.2343

**Published:** 2026-07-01

**Authors:** Juliana Magro

**Affiliations:** 1 juliana.magro@nyulangone.org, Assistant Curator & Undergraduate Medical Education Librarian, NYU Health Sciences Library, NYU Grossman School of Medicine, NYU Langone Health, New York, NY

**Keywords:** Assessment, Medical Education, Evidence-Based Medicine, Medical Librarianship

## Abstract

**Background:**

Librarians providing feedback on Evidence-Based Medicine (EBM) assignments face time constraints. This case report describes implementing a semi-automated rubric — defined here as a tool that automatically calculates scores from grader-selected ratings while narrative feedback is still written by the instructor — to reduce grading time for an EBM capstone assignment for first-year medical students.

**Case Presentation:**

Two cohorts of approximately 100 students completed the same EBM assignment. The 2023 cohort received manual feedback, while the 2024 cohort received feedback using the semi-automated rubric. Grading time was logged for both cohorts. The rubric, adapted from a template, automatically calculates scores based on selected criteria, streamlining the feedback process.

**Conclusions:**

The semi-automated rubric reduced grading time by 30%, from an average of seven minutes per assignment to five minutes. This simple, adaptable intervention can help reduce educator workload, improve feedback timeliness, and enhance assessment consistency. While limited by its single-grader and single institution design, this case report offers practical insights for educators seeking to improve feedback efficiency in EBM and other disciplines.

## BACKGROUND

Librarians participate in educational interventions related to evidence-based medicine (EBM) via curriculum-integrated instruction programs and standalone classes. They often provide feedback to learners on their performance on EBM-specific assignments or Objective Structured Clinical Examination (OSCEs). Librarian feedback may be limited to distinct EBM steps (formulating questions, searching for evidence, critical appraisal) [1–3], or may include the entire EBM process [4]. This is evidenced in the literature, including in a recent study reporting that librarians’ viewed assessment of EBM as an integral component of their role in educating medical students and as part of their professional identity [5].

Providing timely and consistent feedback as part of these classes make consistent feedback implementation difficult—a challenge compounded by institutional and administrative barriers, discussed below. Overall, medical educators face time limitations and resource constraints (clinical scheduling, patient care expectations, limited compensation) that make consistent feedback implementation difficult [6]. Librarians also face these challenges, often being short-staffed and having many other duties beyond teaching and providing feedback on students’ assignments [5]. This is particularly challenging when librarians are providing formative, written feedback to students, which is an onerous activity for all educators [7]. Even librarians who are teaching and assessing students — and, at some level, have structured support from the institution — may struggle with the time-intensive challenge of providing feedback.

In addition to time constraints, health sciences librarians must also navigate institutional and administrative barriers, including the need to develop relationships with leadership to advocate for curricular time and assessment [5]. Educational dynamics in medical schools are complex, and curricular load for EBM varies. Health sciences librarians play an important role in participating in the education of medical students, being involved in curriculum design, implementation, and assessment [8–11]. At the same time, North American medical schools are increasingly adopting accelerated curricula to expedite student progression through the curriculum [12]. In shortened curricula, leadership needs to make strategic decisions about what needs to be kept and what needs to change. It has not yet been reported if these changes have affected librarians teaching in the curriculum, but it is likely that some changes might occur as a result.

In summary, while the incorporation of timely and effective feedback in EBM education is important in equipping students with lifelong learning skills, several challenges must be navigated by librarians. This case report will focus specifically on addressing time constraints in grading assignments and providing feedback.

To aid in this challenge, I developed a semi-automated rubric for an EBM capstone assignment, embedded in the curriculum of first-year medical students at NYU Grossman School of Medicine. This case report narrates the experience of implementing this rubric — described in detail below — with the goal of reducing the time needed to provide feedback to students. My hypothesis is that the use of the rubric would decrease grading time per assignment, therefore alleviating workload. In sharing this experience, I hope to provide insights and strategies that other librarians and educators can use to enhance efficiency in similar educational settings. This report follows the guidelines from Standards for Quality Improvement Reporting Excellence in Education (SQUIRE-EDU) [13] (Appendix A).

## CASE PRESENTATION

### Context

This mandatory assignment was part of a curriculum integrated EBM course for first-year medical students at NYU Grossman School of Medicine, and it was embedded in the learning management system (LMS) as a quiz. As part of the assignment, students were asked to complete all EBM steps in sequence (Ask, Acquire, Appraise, Apply, Assess), based on a patient scenario — this is also called a whole-task assignment [4, 14]. With this assignment, they would practice the individual components of EBM in an integrated manner. Although students received a grade, this was a formative assessment and did not count towards their final course grade. After completion, students were given both a grade and narrative, individual feedback.

This case report analyzed grading time of two cohorts: 2023 and 2024. The assignments for the 2023 cohort were graded without the aid of the semi-automated rubric, while the assignments for the 2024 cohort were graded with the aid of the semi-automated rubric that will be described below in the “intervention” section below.

For both cohorts, several characteristics were the same: the class size (107 for the 2023 cohort, and 102 for the 2024 cohort), the curriculum content and timing, the assignment, and the instructors. The same person graded the assignments for both cohorts.

### Intervention

**Figure 1:**
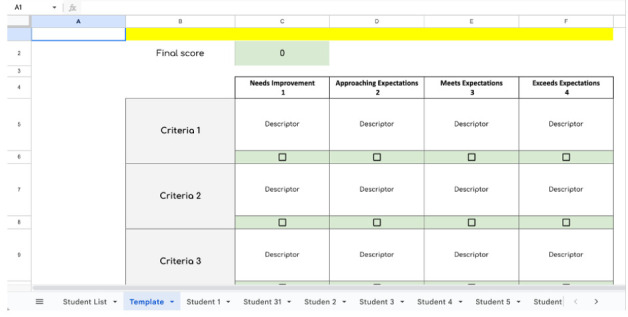
Google Sheets four-point rubric template. The “Template” tab shows example criteria (Criteria 1–3) and four performance levels (Needs Improvement, Approaching Expectations, Meets Expectations, Exceeds Expectations) with editable descriptors and checkboxes for scoring. A “Final score” cell at the top summarizes the selections.

The original rubric template was described and shared by an educational technology specialist [15] and consists of a Google Sheets document with a blank four-point rubric, as shown in Figure 1 below. The template shows different criteria, a space for each criterion to add a description, and several tabs: a student list tab and individual student tabs. Any changes made to the template tab will also apply to all the students' tabs. In this way, one tab can be used for each student.

Using the template as a starting point, I adapted it to fit the rubric I developed for the assignment in 2021^*^.

**Figure 2:**
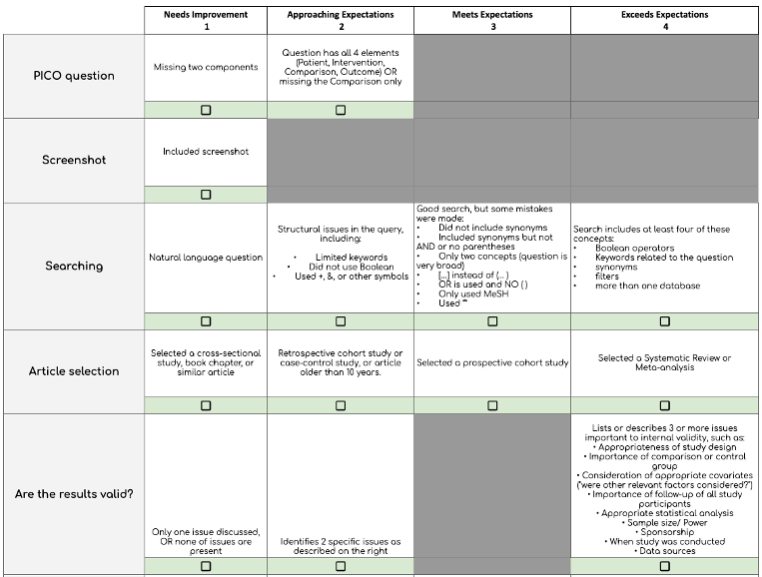
Adapted Google Sheets showing a portion of the rubric. The rubric lists EBM-specific criteria (e.g., PICO question, screenshot, searching, article selection, and validity of results) in rows and four performance levels (Needs Improvement, Approaching Expectations, Meets Expectations, Exceeds Expectations) in columns, each with detailed descriptors and checkboxes to guide scoring.

Adapting the rubric to the Google Sheets format was straightforward and took a few hours, completed in a single work session. This process involved customizing the original rubric template to align with specific learning objectives and assessment criteria of the EBM assignment. Key steps included revising criterion descriptions to reflect EBM proficiency, calibrating grading for the different criteria, and sharing the rubric with other medical educator librarians at the institution for feedback prior to implementation. A snapshot of the adapted rubric can be seen below, in figure 2. The full rubric is also shared online^†^.

In parallel with the rubric development, I built a document containing standardized feedback sentences addressing common learning needs. For example, if students used different symbols (like &, +) to connect keywords in PubMed, or if they mention the results without informing the measure of association or statistical significance, I compiled reusable feedback statements. This allowed me to maintain personalized feedback while improving grading efficiency for common patterns.

**Figure 3:**
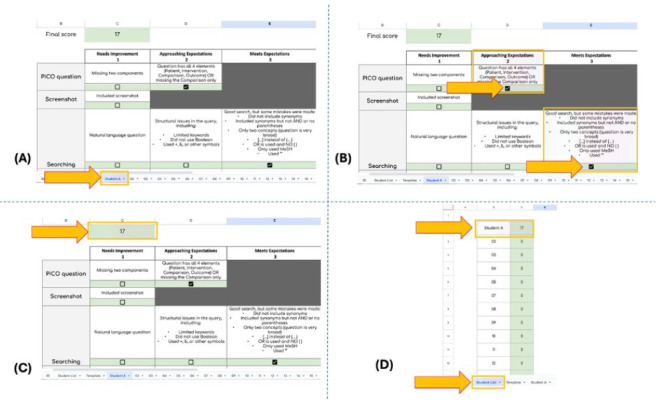
Example workflow for using the rubric in Google Sheets. (A) The assessor selects an individual student tab (e.g., “Student A”). (B) The appropriate checkbox is marked for each criterion to reflect the student’s performance. (C) The overall “Final score” is calculated automatically on the student’s rubric tab. (D) In the “Student List” tab, the student’s name and final score are automatically updated, creating a cumulative list of graded assignments.

The step-by-step use of this rubric is illustrated in Figure 3. First, one can enter the name of the student they are grading for a particular tab, for example “Student A” (A). Next, the assessor can checkmark the box that best matches the student performance (B). This will lead to an automatic score, as shown in (C). Finally, in the first tab named “Student list,” the name and grade of the student is automatically updated (D), allowing for assessors to quickly gather a list of all graded assignments.

Following the grading step, I would provide narrative feedback by selecting and adapting sentences from the standardized feedback document as needed. Thus, the rubric handles the scoring aspect automatically but still requires human input for personalized feedback, which is why I refer to this rubric as semi-automated. Appendix B shows an example of one graded assignment feedback (preserving the student submission).

### Measures and Results

For both cohorts, I kept a log of total time spent assessing student assignments. This log contained the date, the length of the work session, and how many assignments were graded in that session (for example, 3/21/2024, 10 assignments, 51 min). Time was tracked using a digital stopwatch app, noting start and end times. A session is described as a focused and uninterrupted block of time where the only activity performed by the author was grading. To minimize interruptions, email and work chat applications were closed, and the phone was put in silent mode. If an interruption occurred mid-grading, the timer was paused. If a break was needed, the session was ended and a new session was started after returning to grading. Brief transitions between assignments (e.g., opening the next file) were included in the session time. Appendix C contains the data logs for both cohorts.

With the log for the manual feedback and the semi-automated feedback cohorts, I measured the average grading time for each assignment, and the difference between each cohort. The table below shows the results for each cohort.

**Table 1:** Results

	*Manual feedback (2023)*	*Semi-automated feedback (2024)*
***Assignments (n)***	80	69
***Time (min)***	561	353
***Average time per assignment (min)***	7	5

As shown in the table above, for the 2023 cohort (manual feedback), 80 students completed the assignment at the time of grading, and the average grading time was seven minutes. For the 2024 cohort (semi-automated feedback), 69 students completed the assignment at the time of grading, and the average grading time was five minutes. These results show a 30% reduction in grading time with the use of this rubric.

### Ethical considerations

According to the NYU Langone Health IRB, this study constitutes a quality improvement project and is, therefore, exempt from review. No student-specific data was collected.

## DISCUSSION

The key finding of this case report was that there was a 30% reduction in grading time with the semi-automated rubric. This is a low-cost, easy to adapt, and easy to implement intervention which can result in time saved when grading assignments and quizzes, helping reduce educator workload. Streamlining grading can, in turn, help alleviate some of the institutional and administrative barriers related to time constraints, as discussed in the background section.

This intervention may also benefit students. By decreasing the workload, educators can hopefully provide feedback in a timely manner so that students can use that feedback to improve performance. Another potential benefit is ensuring consistency of assessment across students and cohorts. However, no outcomes related to student perception were measured in this implementation and warrant future investigation.

An important consideration when implementing new tools is their sustainability. Since this is a Google Sheets format, it is easy to maintain, and it can be shared with fellow educators with ease. It is advisable to review the content and update it, if necessary, prior to reusing the rubric. Fortunately, with the template format, all student tabs are updated automatically, simplifying the process.

The potential for adapting and implementing similar automated rubrics extends beyond EBM courses to other disciplines. Automated rubrics can enhance efficiency in grading across various educational settings, including nursing, pharmacy, and other health science courses.

There are limitations to this case report, as it was not structured or conducted as empirical research. The primary limitation is the reliance on a single grader's experience, which may not generalize across different educators or institutional contexts. Different educators may have different baseline grading speeds, rubric interpretation approaches, and feedback styles, meaning that the 30% reduction observed may not be representative of other educators’ experience. With this, it is difficult to affirm that these differences would remain in the same magnitude in other scenarios.

One potential confound is that grading efficiency improvements could result from the grader becoming more familiar with the assignment rather than the assistance of the rubric. However, I argue that this is unlikely to be a significant factor in this case: the assignment was first created in 2021 and had been used for two prior cohorts before the control cohort in 2023. By the time of the control cohort assessment, I had already graded this specific assignment over two hundred times. While some minimal practice effects cannot be completely ruled out, the magnitude of the efficiency improvement observed (30% reduction) is unlikely to be explained by familiarity gains alone.

This case report measured only grading time reduction and did not assess student outcomes such as time to feedback delivery, student satisfaction with feedback, perceived consistency or helpfulness of feedback, or impact on performance after the assignment. While consistency is a theoretical benefit of standardized rubrics, I did not measure whether feedback was more consistent across students or across cohorts. In addition, this rubric was designed for a structured, whole-task EBM assignment for preclinical students. The time savings may not generalize to different assignments or different formats (e.g., presentations, clinical evaluations).

Future research on this subject could focus on assessing different assignments or quizzes with multiple graders to establish inter-rater reliability. Researchers should consider other outcomes, such as student satisfaction with feedback (including whether the combination of standardized and personalized elements affects perceived utility, personalization, or satisfaction) student performance on subsequent assignments, or faculty satisfaction with the assessment process. The initial time investment required to develop and calibrate similar rubrics should also be documented to provide a more complete picture of the process. Finally, future studies should incorporate larger sample sizes and diverse educational settings, in addition to tracking individual assignment grading time to assess where automation is most or least useful.

## Data Availability

Data reported in this article are provided in Appendix C.

## Footnotes

* As the rubric was developed before planning this documentation, specific details about different iterations, and the rubric development process were not recorded.

† Adapted Google Sheets Rubric: https://bit.ly/2025-MLA
