# Supplementary material for: Semi-automated rubrics for evidence-based medicine assessment: a case report on grading time reduction
Source: J Med Libr Assoc. 2026 Jul 14;114(3):290–6. doi: 10.5195/jmla.2026.2343 (PMC13367300; doi:10.5195/jmla.2026.2343)
Supplement: Supplementary file 1 — Appendix A [file jmla-114-3-290-s01.pdf]

Appendix A: SQUIRE-EDU checklist

	SQUIRE-EDU item	*Text in the manuscript*	*Comments*
1. Title	EDU 1: Indicate that the manuscript concerns efforts to improve health professions education systems and learning.	Automated Rubrics for Evidence-Based Medicine Assessment: A Case Report on Grading Time Reduction	Indicates the focus on educational improvement.
2. Abstract	EDU 2: Keywords include a focus on education and learning.	Medical education, Evidence-Based Medicine, Assessment	Keywords focus on education.
3. Problem Description	EDU 3: Description of the nature and significance of the need for change in the local educational system.	Providing timely and consistent feedback […] poses a significant challenge related to time constraints and workload. Institutional and administrative barriers play a role in how feedback is provided and received. Overall, medical educators face time limitations and resource constraints (clinical scheduling, patient care expectations, limited compensation) that make consistent feedback implementation difficult [6]. Librarians also face these challenges, being often short-staffed and having many other duties beyond teaching and providing feedback on students' assignments [5]. This is particularly challenging when	Describes the problem of time constraints in providing feedback.

		librarians are providing formative, written feedback to students, which is an onerous activity for all educators [14]. Even librarians who are teaching and assessing students — and, at some level, have structured support from the institution — may struggle with the time-intensive challenge of providing formative, written feedback that supports learning.<br>[…] While the incorporation of timely and effective feedback in EBM education is important in equipping students with lifelong learning skills, several challenges must be navigated by librarians. This case report will focus specifically addressing time constraints in formative feedback. To aid in this challenge, I developed a semi-automated rubric for an EBM capstone assignment, embedded in the curriculum of first-year medical students at [School].	
5. Rationale	EDU 5: Identify the guiding theory (learning, change, implementation, or other) and how it aligns with the need for	This case report narrates the experience of implementing this rubric — described in detail below — with the goal of reducing the time needed to provide feedback to students.	Rationale for implementing the rubric is provided

	change in the local educational system.		
7. Context	EDU 7a: Contextual elements for learning (e.g. setting, program, people, resources, social, geopolitical influences) before the intervention(s).	This mandatory assignment was part of an EBM course embedded in the curriculum of first-year medical students at [School]...For both cohorts, several characteristics were the same: the class size (~100 students), the curriculum content and timing, the assignment, and the instructors. The same person graded the assignments for both cohorts.	Describes the context of the EBM course, student cohort, and assignment.
8. Intervention(s)	EDU 8a: Description of the primary intervention(s) and co-interventions (e.g. faculty or tool development).	The original rubric template was described and shared by an educational technology specialist...Figures 2 and 3 help illustrate the adaptation process.	Describes the development and implementation of the semi-automated rubric.
9. Study of the Interventions	EDU 9a: Approach used to understand the impact of the educational intervention(s) on the learner and beyond, such as impact on patients, families, the community, faculty, educational programs, or the healthcare system.	For both cohorts, I kept a log of assessment time session. This log contained the date, the length of the work session, and how many assignments were graded in that session (for example, 3/21/2024, 10 assignments, 51 min). A session here is described as a focused and uninterrupted block of time where the only activity performed by the author was grading. Appendix 2 contains the data logs for both cohorts.	Describes the data collection method (grading logs), and provides the data.

10. Measures	EDU 10: Quantitative and/or qualitative measures chosen to assess the educational processes and outcomes on learners, faculty, educational programs, patients, families, healthcare systems, or communities.	With the log for the manual feedback and the semi-automated feedback cohorts, I measured the average grading time for each assignment, and the difference between each cohort.	Specifies the measure used (average grading time).
12. Ethical Considerations	EDU 12: Approaches to address vulnerability of learner participants.	According to the NYU Langone Health IRB, this study constitutes a quality improvement project and is, therefore, exempt from review. No student-specific data was collected.	Addresses ethical considerations.
13. Results	EDU 13: For each educational intervention and co-intervention, provide details about iterative modifications based on the assessment of the learning.	As shown in the table above…These results show a 30% reduction in grading time with the use of this rubric.	Presents the results of the intervention, including the reduction in grading time
14. Summary	EDU 14: Connect the findings to the guiding theory (learning, change, implementation, other) used to direct the change in the local educational system.	The key finding of this case report was that there was a 30% reduction in grading time with the automated rubric. This is a low-cost, easy to adapt, and easy to implement intervention which can result in time saved when grading assignments and quizzes, helping reduce educator workload.	Summarizes the findings and connects them to the rationale

15. Interpretation	EDU 15: Include the impact of the intervention(s) on learners, faculty, educational program, patients, families, healthcare systems, or communities.	Streamlining grading can, in turn, help alleviate some of the institutional and administrative barriers related to time constraints, as discussed in the background section… ensuring consistency of assessment across students and cohorts.	Discusses the impact of the intervention on workload and consistency
17. Conclusions	EDU 17b: Scalability of the work to other learners and contexts.	The potential for adapting and implementing similar automated rubrics extends beyond EBM courses to other disciplines…Future studies should also incorporate larger sample sizes and diverse educational settings.	Discusses the scalability and generalizability of the findings
