# Supplementary material for: Semi-automated rubrics for evidence-based medicine assessment: a case report on grading time reduction
Source: J Med Libr Assoc. 2026 Jul 14;114(3):290–6. doi: 10.5195/jmla.2026.2343 (PMC13367300; doi:10.5195/jmla.2026.2343)
Supplement: Supplementary file 2 — Appendix B [file jmla-114-3-290-s02.pdf]

Appendix B: Example submission

For this example, the student's responses were redacted. The graded scores were generated by using the automated rubric and the individual feedback, when provided, was copied and pasted from the standardized feedback document, and adapted as needed (these items are shown in blue).

EBM STEP 1: ASK

Scenario for reference:

Your patient is 17 years old, and recently has been struggling with suicidal ideation. They are a recreational marijuana user with no history of mental health disorders. You are wondering whether marijuana use might be associated with suicidal thoughts and behavior.

Question 1: Create a PICO question for this case:

For a teenager with no history of mental health disorders, is recreational marijuana use, compared to no marijuana use, associated with suicidal thoughts or behavior?

[student response redacted]

Score: 2/2

EBM STEP 2: ACQUIRE

Question 2: Take a screenshot of the **advanced search page** of the databases(s) searched and attach to this question.

Score: 1/1

Question 3**:** List your final search strategy (keywords AND filters used, if any). Make sure that your keyword list matches the screenshot you provided above, including parentheses, Boolean operators, etc.

[student response redacted]

Score: 3/4

Feedback

Good search! Something to consider when you are using quotes is that you lose automatic mapping to MeSH terms. Whether that will make a difference or not, it will depend on the search. But take a look below at a search with quotation marks and one without. The one without phrase searching maps to cannabis [MeSH].

keyword: marijuana
PubMed will search for: "cannabis"[MeSH Terms] OR "cannabis"[All Fields] OR "marijuana"[All Fields] OR "marijuana s"[All Fields]

keyword: "marijuana"
PubMed will search for: "marijuana"[All Fields]

When you do not use MeSH, you might miss certain articles that are not using the keywords you chose, but are indexed under those MeSH terms.

Question 4**:** Select one article to answer your question (include the citation and a link the article)

[student response redacted]

Score: 4/4

Feedback

Well done selecting a recent systematic review related to this topic!

EBM STEP 3: APPRAISE

In this section you will appraise the study you found.

You may select the appropriate CASP checklist to help guide the appraisal. https://casp-uk.net/casp-tools-checklists/

Question 5: Are the results of the study valid?

[student response redacted]

Score: 4/4

Feedback

In addition to what you point out, this review seems to have other shortcomings that could introduce bias:

- Search included grey literature and unpublished studies, but authors only included peer-reviewed articles in their analysis, which automatically excluded the results from those searches;
- There is mention of quality assessment, but the results of this assessment are not presented in the article or in the appendix. In other words, it is not clear what the risk of bias is in the studies included, and how they informed conclusions or not;
- For the studies included for the outcome of suicidal ideation, authors do not describe what the

total population of those studies is and, again, the risk of bias. These are important considerations to ensure a more rigorous meta-analysis.

- the authors stated that when one study provided an odds ratio for many different frequencies of marijuana use, they only included the highest odds ratio. This could push the results of the meta-analysis towards a higher odds ratio.

Other possible limitations include the fact that the included studies did not control for socioeconomic factors, concurrent drugs use, quantity of marijuana consumed, or other psychosocial factors that could contribute to early marijuana use.

Question 6: What are the results?

[student response redacted]

Score: 2/4

Feedback

When you are reporting results to your attending, make sure to mention the measure of association (and statistical significance too!).

Question 7: Will the results help locally?

[student response redacted]

Score: 4/4

EBM STEP 4: APPLY

Question 8: Will you apply the evidence you found to your case? Briefly explain your decision below:

[student response redacted]

Score: 1/1

EBM STEP 5: ASSESS

Question 9: Evaluate your performance. Ask yourself a few questions about this EBM cycle, for example:

- Did you ask an answerable clinical question?
- Did you find the best evidence available?
- Did you find the most current evidence available?
- Did you critically appraise the evidence?

- Did you evaluate the evidence for its internal and external validity?
- Should you modify your clinical question and start over?

Score: 1/1
