# Supplementary material for: Semi-automated rubrics for evidence-based medicine assessment: a case report on grading time reduction
Source: J Med Libr Assoc. 2026 Jul 14;114(3):290–6. doi: 10.5195/jmla.2026.2343 (PMC13367300; doi:10.5195/jmla.2026.2343)
Supplement: Supplementary file 3 — Appendix C [file jmla-114-3-290-s03.pdf]

Appendix C: Data logs

Table 1: 2024 cohort (with semi-automated rubric)[1]

Date	*Number of assignments graded*	*Time (min)*
No date	5	27
No date	5	29
No date	3	25
No date	5	25
3/21/2024	10	51
3/21/2024	10	50
3/22/2024	11	50
3/22/2024	10	50
3/25/2024	6	26
3/25/2024	4	20
Total	**69**	**353**

Table 2: 2023 cohort (without semi-automated rubric)

Date	*Number of assignments graded*	*Time (min)*
6/28/2023	4	39
6/28/2023	7	54
6/28/2023	7	57
6/28/2023	8	51

[1] Four early grading sessions were not dated during initial data collection; dates were recorded consistently beginning 3/21/2024.

6/29/2023	5	34
6/29/2023	9	67
6/29/2023	8	50
6/29/2023	9	39
6/29/2023	2	15
6/30/2023	4	29
6/30/2023	9	65
6/30/2023	8	61
Total	**80**	**561**
